# A Case of a Stafne Bone Defect Associated with Sublingual Glands in the Lingual Side of the Mandible

**DOI:** 10.1155/2020/8851174

**Published:** 2020-12-17

**Authors:** Kamichika Hayashi, Takeshi Onda, Takahiro Iwasaki, Mitsuru Takata, Kiyotaka Mori, Hiroyuki Matsuda, Shinya Watanabe, Hidetoshi Tamura, Takahiko Shibahara, Masayuki Takano

**Affiliations:** ^1^Department of Oral and Maxillofacial Surgery, Tokyo Dental College, Tokyo, Japan; ^2^Oral and Maxillofacial Surgery, Kameda General Hospital, Chiba, Japan

## Abstract

A Stafne bone defect from the mandibular anterior to the premolar region is an extremely rare case. A case of a Stafne bone defect extending from the mandibular anterior to the premolar region was presented. Computed tomography (CT) and magnetic resonance imaging (MRI) suggested that salivary gland tissue connected to the sublingual glands was involved in the formation of the cavity. The patient was a 68-year-old man who was examined at our hospital's emergency outpatient department after a traffic accident. He was referred to our department for the treatment of contusions of the lips and oral cavity. A bone defect in the lingual side of the mandible from the right anterior to the right premolar region was incidentally detected on CT. CT showed a rounded cavity in the lingual side of the mandible that had a lingual opening, was monocystic, and had a cortical margin. The margin of the cavity was relatively dull and regular. MRI showed that the tissue filling the cavity in the lingual side of the mandible had similar signal intensity as the sublingual glands and was contiguous with the normal sublingual glands. Based on these findings, the bone defect was diagnosed as a Stafne bone defect filled with salivary gland tissue connected to the sublingual gland tissue.

## 1. Introduction

The first case of a Stafne bone defect was reported by Stafne in 1942 [1]. Stafne bone defect are also known as the so-called Stafne bone cavity or static bone defect [2, 3]. A Stafne bone defect is a cavity in the lingual side of the mandible in the vicinity of the mandibular angle that is filled with salivary gland tissue, adipose tissue, lymphatic tissue, or other tissues. It is generally asymptomatic and is usually incidentally detected by oral-maxillofacial radiography [2]. Radiography depicts this condition as a unilocular radiolucent area with a clear boundary and a smooth margin. It is sometimes suspected to be a cyst or tumor in the jawbone [4]. Usually, a Stafne bone defect is found inferior to the mandibular canal from the mandibular molars to the mandibular angle [5]. A case with a Stafne bone defect from the mandibular anterior to the premolar region that appeared to be filled with salivary gland tissue connected to the sublingual glands is reported, along with a short discussion of the literature.

## 2. Case Presentation

Fully informed consent for publication of clinical information relating to this case was obtained from the patient.

The patient was a 68-year-old man who was examined at our hospital's emergency outpatient department after his face was struck by the steering wheel during a traffic accident. The emergency room doctor performed computed tomography (CT), which showed no abnormalities of the internal organs or brain. That same day, he was referred to our department for the treatment of contusions of the lips and oral cavity. No abnormal findings in his medical history or family history were noted. When he arrived at the hospital, he was in pain and bleeding from the contusions, but there were no signs of nerve paralysis or infection. The contusions of the lips and oral mucosa were debrided and sutured.

**Figure 1:**
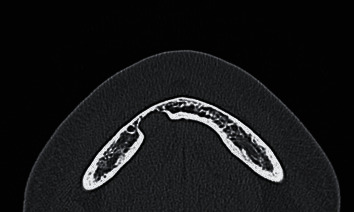
Computed tomography image, horizontal cross section, hard tissue mode. A rounded cavity is observed in the lingual side of the mandible from the right anterior to the premolar region. The margin of the cavity in the lingual side of the mandible is a high-density line, suggesting cortical bone, and is smooth. The mandibular canal is not connected with the cavity.

**Figure 2:**
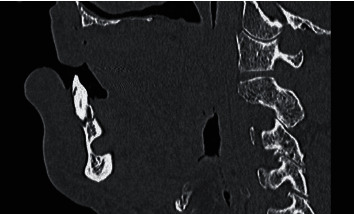
Computed tomography image, sagittal cross section, hard tissue mode. The lingual cortical bone recessed from the lingual to the buccal side is contiguous with the buccal cortical bone at the deepest point of the cavity. There is no connection of the apex of the adjacent #27 with the cavity in the lingual side of the mandible, and a high-density line suggests the presence of bony tissue between the apex of #27 and the cavity in the lingual side of the mandible.

**Figure 3:**
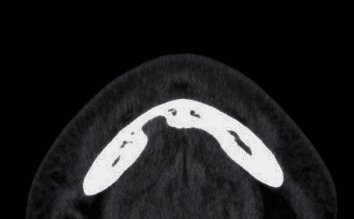
Computed tomography (CT) image, horizontal cross section, soft tissue mode. The inside of the cavity in the lingual side of the mandible has similar soft tissue density (CT number, 40–70 Hounsfield units (HU)) to the sublingual glands (CT number, 40–70 HU), with findings that suggest continuity with the sublingual glands.

The CT performed in the emergency department showed no fractures anywhere. However, it showed a cavity in the lingual side of the mandible from the right anterior to the premolar region. CT in hard tissue mode showed a high-density line, suggesting cortical bone on the margin of the cavity in the lingual side of the mandible (Figure 1). The lingual cortical bone was recessed from the lingual side to the buccal side and, at the most recessed point, was contiguous with the buccal cortical bone (Figure 2). There was no continuity between the apex of the adjacent #27 and the cavity in the lingual side of the mandible, and a high-density line suggested the presence of bony tissue between the apex of #27 and the cavity in the lingual side of the mandible. There was no continuity between the cavity in the lingual side of the mandible and the mandibular canal. CT in soft tissue mode showed that the inside of the cavity in the lingual side of the mandible had similar soft tissue density (CT number, 40–70 Hounsfield units (HU)) to the sublingual glands (CT number, 40–60 HU) and was contiguous with the sublingual glands (Figure 3). Based on these CT findings, our presumed diagnosis was a Stafne bone defect.

**Figure 4:**
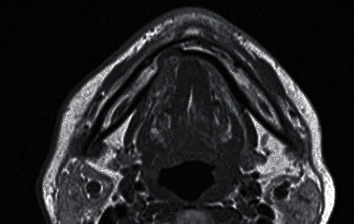
Magnetic resonance imaging, horizontal cross section, T1-weighted imaging. A rounded cavity is observed in the lingual side of the mandible from the right anterior to the premolar region. The margin of the cavity in the lingual side of the mandible exhibits a cortical bone structure, and the cavity's margin is smooth. A layer of myeloid tissue is observed in the cavity between the lingual cortical bone and the buccal cortical bone. The tissue in the cavity in the lingual side of the mandible has similar signal intensity to the sublingual glands and is contiguous with the sublingual gland tissue.

**Figure 5:**
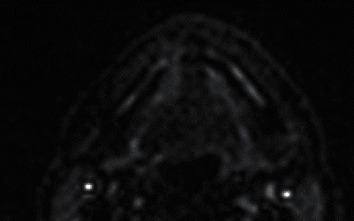
Magnetic resonance imaging, horizontal cross section, short T1 inversion recovery. There are no signs of tumors or inflammation.

In 3 months after the first examination, magnetic resonance imaging (MRI) was performed to examine the tissue inside the cavity in the lingual side of the mandible. The area exhibited greater hyperintensity than the muscle and similar signal intensity to the sublingual gland tissue on T1-weighted and T2-weighted images and was filled with soft tissue contiguous with the sublingual glands. Short T1 inversion recovery images showed no signs of tumors or inflammation in the surrounding area. A final diagnosis of a Stafne bone defect was established based on the presence of a cavity in the lingual side of the mandible from the right anterior to the premolar region that was filled with salivary gland tissue connected to the sublingual gland tissue (Figures 4 and 5). Subsequently, regular imaging was performed, and the patient was followed up.

Abnormal findings had not been observed 12 months after the first examination.

## 3. Discussion

In general, static bone cavities are often observed near the mandibular angle starting posterior to the mandibular molars. In panoramic radiographs, the cavity usually appears as a single cystic bone defect with a distinct round boundary surrounded by radiolucent tissue. Cavities are often detected incidentally [5]. The probability rate of incidentally discovering a Stafne bone defect in panoramic radiography is 0.08%–0.7%, which is predominantly observed in men in their 40s or 50s [4]. In addition, static bone cavities are less common in the front teeth and premolar areas. The probability rate of discovering a monocystic radiolucent area with a distinct boundary around the front teeth or premolars in panoramic radiography is 0.009% [5]. Schneider proposed a diagnostic algorithm for static bone cavities in panoramic radiographs, but this is not considered suitable for static bone cavities around the anterior region because of the narrow tomographic area in panoramic radiographs in this area, the overlapping shadows of the cervical spine, and other factors [6].

CT and MRI are useful for diagnosing static bone cavities^2^. CT is particularly effective for morphological evaluations of the mandible. Some studies have considered CT to be the most effective tool for diagnosing static bone cavities [7], whereas others have found that CT is insufficient and MRI is the most effective tool [8]. CT is generally the first choice if abnormal findings are observed in radiographs [9]. CT findings of a Stafne bone defect include a rounded cavity in the lingual side of the mandible that has a lingual opening, is monocystic, and has a cortical margin. The margin of the cavity is relatively dull and regular. A Stafne bone defect can be diagnosed when these findings are observed, and no additional tests are considered necessary [5, 10]. CT-sialography is more useful than plain CT when investigating connections with the major salivary glands [11]. However, because it is difficult to selectively contrast the sublingual glands to examine associated lesions, CT-sialography is considered inappropriate.

Compared to CT, MRI involves no radiation exposure and has higher histological resolution and is thus suitable for depicting the properties of tissue filling the cavity in the lingual side of the mandible [12, 13]. MRI is particularly useful for examining tissue filling the cavity in the lingual side of the mandible. If MRI suggests that the tissue is normal, additional tests that are surgically invasive can be avoided [12]. Furthermore, MRI can visualize the cortical bone and marrow of the mandible. In the present case, it was confirmed that the cavity in the lingual side of the mandible had a cortical margin, and because the signal intensity of the tissue in the cavity in the lingual side of the mandible was similar to that of the sublingual gland tissue, the presence of a tumorous lesion was unlikely, and it was considered normal salivary gland tissue. Combining CT and MRI improves the reliability in diagnosing a Stafne bone defect [2].

The exact pathogenesis has not been clarified yet. Previous theories of the origins of static bone cavities include those of Fordyce [14], Rushton and Cantab [15], Thoma [16], Choukas and Toto [3], and Harvey and Noble [17]. A Stafne bone defect is currently considered to occur when postnatal mandibular bone absorption creates a cavity due to pressure from salivary gland tissue and the surrounding soft tissue [5, 8, 12, 18]. That is, the most widely accepted view is that the cavities develop as a result of a localized pressure atrophy of the lingual surface of the mandible from the adjacent salivary gland [19]. In the present case, both CT and MRI showed recessed sublingual gland tissue contiguous with the salivary gland tissue from the mandibular anterior to the premolar region, indicating that the Stafne bone defect formed postnatally due to pressure from the sublingual gland tissue in the lingual side of the mandible.

The differential diagnostic of Stafne bone defect includes odontogenic cystic, salivary gland tumors, fibro-osseous lesions, central giant cell lesions, hyperparathyroidism, ameloblastoma, eosinophilic granuloma, hemangioma of the bone, myxoma, aneurismal bone cyst, multiple myeloma, and benign neurogenic tumors [4, 19, 20].

In the present case, CT showed a rounded cavity in the lingual side of the mandible that had a lingual opening, was monocystic, and had a cortical margin. The margin of the cavity was relatively dull and regular, and the CT number of the tissue filling the cavity in the lingual side was similar to that of the sublingual glands. As was observed on CT, MRI also showed a rounded cavity in the lingual side of the mandible that had a lingual opening, was monocystic, and had a cortical margin, with a relatively dull and regular margin. MRI also confirmed that the signal intensity of the tissue inside the cavity in the lingual side of the mandible was similar to that of the sublingual glands, that the cavity was filled with tissue contiguous with the normal sublingual glands, and that tumors or inflammatory findings were not observed in the surrounding area. Based on these CT and MRI findings, a final diagnosis of a Stafne bone defect with a cavity in the lingual side of the mandible from the right anterior to the premolar region that was filled with salivary gland tissue connected to the sublingual gland tissue was established.

Static bone cavities do not require aggressive treatment in the absence of functional disorders or subjective symptoms. Clinicians rarely perform additional imaging or surgical procedures on cases that are diagnosed by CT or MRI and usually opt for follow-up. Although biopsies and other surgically invasive tests are important for establishing a definitive diagnosis, considering the pathology of static bone cavities, performing thorough imaging examinations and establishing a diagnosis noninvasively should be prioritized. Whereas static bone cavities are rare, there have been reports of an increasing trend [21] and of detecting pleomorphic adenoma in the cavity area [22]. Therefore, the determination of stasis in a case diagnosed as Stafne bone defect should not be made based on a single time point, but through follow-up observations. In either case, surgical treatments can be selected if follow-up shows that the shape of the Stafne bone defect is increasing in size or that the properties of the tissue filling the cavity in the lingual side of the mandible are changing. Data on more cases need to be accumulated to determine the proper intervals and durations for follow-up.

## 4. Conclusion

A case of a Stafne bone defect extending from the mandibular anterior to the premolar region was presented. CT and MRI suggested that salivary gland tissue connected to the sublingual glands was involved in the formation of the cavity.

## References

[1] Stafne E. C. (1990). Bone cavities situated near the angle of the mandible. *The Journal of the American Dental Association*.

[2] Schneider T., Filo K., Locher M. (2014). Stafne bone cavities: systematic algorithm for diagnosis derived from retrospective data over a 5-year period. *British Journal of Oral and Maxillofacial Surgery*.

[3] Choukas N. C., Toto P. D. (1960). Etiology of static bone defects of the mandible. *Journal of Oral Surgery, Anesthesia, and Hospital Dental Service*.

[4] Assaf A. T., Solaty M., Zrnc T. A. (2014). Prevalence of Stafne’s bone cavity--retrospective analysis of 14,005 panoramic views. *In Vivo*.

[5] Philipsen H. P., Takata T., Reichart P. A., Sato S., Suei Y. (2002). Lingual and buccal mandibular bone depressions: a review based on 583 cases from a world-wide literature survey, including 69 new cases from Japan. *Dentomaxillofacial Radiology*.

[6] Venkatraman S., Gowda J. S., Kamarthi N. (2011). Unusual ghost image in a panoramic radiograph. *Dentomaxillofacial Radiology*.

[7] Quesada-Gómez C., Valmaseda-Castellón E., Berini-Aytés L., Gay-Escoda C. (2006). Stafne bone cavity: a retrospective study of 11 cases. *Medicina Oral Patologia Oral Y Cirugia Bucal*.

[8] He J., Wang J., Hu Y., Liu W. (2019). Diagnosis and management of Stafne bone cavity with emphasis on unusual contents and location. *Journal of Dental Sciences*.

[9] Kuroda M., Ohmori K., Yamasaki M. (1993). A case of static bone cavity. *Oral Radiology*.

[10] Ariji E., Fujiwara N., Tabata O. (1993). Stafne’s bone cavity: classification based on outline and content determined by computed tomography. *Oral Surgery, Oral Medicine and Oral Pathology*.

[11] Baker G. (1988). A radiolucency of the ascending ramus of the mandible associated with invested parotid salivary gland material and analogous with a Stafne bone cavity. *British Journal of Oral and Maxillofacial Surgery*.

[12] Branstetter B. F., Weissman J. L., Kaplan S. B. (1999). Imaging of a Stafne bone cavity: what MR adds and why a new name is needed. *American Journal of Neuroradiology*.

[13] Minowa K., Inoue N., Sawamura T., Matsuda A., Totsuka Y., Nakamura M. (2003). Evaluation of static bone cavities with CT and MRI. *Dentomaxillofacial Radiology*.

[14] Fordyce G. L. (1956). The probable nature of so-called latent haemorrhagic cysts of the mandible. *British Dental*.

[15] Rushton M. H., Cantab B. C. (1946). Solitary bone cysts in the mandible. *British Dental Journal*.

[16] Thoma K. H. (1955). Case report of a so-called latent bone cyst. *Oral Surgery, Oral Medicine and Oral Pathology*.

[17] Harvey W., Noble N. W. (1968). Defects on the lingual surface of the mandible near the angle. *British Journal of Oral Surgery*.

[18] Sisman Y., Miloglu O., Sekerci A. E., Yilmaz A. B., Demirtas O., Tokmak T. T. (2012). Radiographic evaluation on prevalence of Stafne bone defect: a study from two centres in Turkey. *Dentomaxillofacial Radiology*.

[19] Münevveroğlu A. P., Aydın K. C. (2012). Stafne bone defect: report of two cases. *Case Reports in Dentistry*.

[20] Turkoglu K., Orhan K. (2010). Stafne bone cavity in the anterior mandible. *Journal of Craniofacial Surgery*.

[21] Shibata H., Yoshizawa N., Shibata T. (1991). Developmental lingual bone defect of the mandible. Report of a case. *Journal of Oral and Maxillofacial Surgery*.

[22] Simpson W. (1965). A Stafne’s mandibular defect containing a pleomorphic adenoma: report of case. *Journal of Oral Surgery*.

